# Microporous carbons derived from melamine and isophthalaldehyde: One-pot condensation and activation in a molten salt medium for efficient gas adsorption

**DOI:** 10.1038/s41598-018-24308-z

**Published:** 2018-04-17

**Authors:** Adeela Rehman, Soo-Jin Park

**Affiliations:** Department of Chemistry, Inha University, 100 Inharo, Incheon, 22212 Korea

## Abstract

In the present work, mixture of melamine and isophthalaldehyde undergo simultaneous polymerization, carbonization, and *in situ* activation in the presence of molten salt media through a single all-in-one route to design microporous carbons with high specific surface areas (~3000 m^2^/g). The effect of the activation temperature and molten salts on the polymerization process and final texture of the carbon was explored. Carbon materials prepared at 700 °C, in the presence of KOH (referred as MIK-700), exhibited a narrower pore-size distribution ~1.05 nm than those prepared in the presence of the eutectic KOH-NaOH mixture (MIKN). Additionally, MIK-700 possesses an optimum micropore volume (1.33 cm^3^/g) along with a high nitrogen content (2.66 wt%), resulting in the excellent CO_2_ adsorption capacity of 9.7 mmol/g at 273 K and 1 bar. Similarly, the high specific area and highest total pore volume play an important role in H_2_ storage at 77 K, with 4.0 wt% uptake by MIKN-800 (specific surface area and pore volume of 2984 m^2^/g and 1.98 cm^3^/g, respectively.) Thus, the facile one-step solvent-free synthesis and activation strategy is an economically favorable avenue for designing microporous carbons as an efficient gas adsorbents.

## Introduction

The energy costs related to the separation and sanitization of industrial commodities, such as gases, currently presents approximately 15% of global energy production, and the requirement of such commodities is anticipated to be triple by 2050^1^. Today the need of innovating effective separation and purification methods with lesser energy footprints is more for carbon dioxide (CO_2_) than for any other gas. This is probably due to its contribution to climate change as well as the contamination of other gas streams including natural gas, biogas and syngas^2^. Apart from CO_2_ adsorption and separation, the storage of H_2_, a next-generation energy carrier with a high chemical energy (39 kW h/kg) and ability to provide pollution-free ignition is also need of the present time. Recently, porous carbons are considered as an emerging candidates as H_2_ storage materials by virtue of their facile preparation, low cost, light weight, fast kinetics, and high surface area^3,4^.

Different methods have been reported to fabricate microporous carbon materials with a high surface area^5–7^. Microporosity and high nitrogen content render Schiff-base polymers a highly interesting class of materials for the synthesis of carbons via pyrolysis. For the polymer synthesis, an organic media is used as the solvent for the reaction between primary amines as nucleophilic center and electrophilic carbons of the carbonyl groups to synthesize the polymer framework^8,9^. The resulting carbons obtained by the pyrolysis of the polymers possess a moderate surface area of 500–1500 m^2^/g which can be improved further using KOH as the chemical activating agent. Although chemical activation by KOH is a widely used technique to produce a porous network^10^, the number of variables in the experimental parameters and the differences in the reactivity of precursors limits a comprehensive understanding of the mechanism. In general, the reaction between carbon and KOH begins with the solid–solid interaction followed by the solid–liquid reaction which may reduce the potassium compound to metallic potassium (K), oxidize carbon to carbon oxides and carbonates accompanied by the formation of various active intermediates^11^. However, experimental and theoretical data present a general reaction between carbon and KOH, as presented in Eq. (1).

1 $$6{\rm{KOH}}+2{\rm{C}}\to 2{\rm{K}}+3{{\rm{H}}}_{2}+2{{\rm{K}}}_{2}{{\rm{CO}}}_{3}$$

KOH is entirely consumed when carbon is heated at ~600 °C. At temperatures higher than 700 °C, the as-formed K_2_CO_3_ starts to decompose into CO_2_ and K_2_O and finally vanishes at 800 °C. At temperatures above 700 °C, potassium compounds including K_2_O and K_2_CO_3_ are reduced to metallic potassium, which remains embedded in the carbon matrix.

2 $${{\rm{K}}}_{2}{{\rm{CO}}}_{3}\to {{\rm{K}}}_{2}{\rm{O}}+{{\rm{CO}}}_{2}$$

3 $${{\rm{CO}}}_{2}+{\rm{C}}\to 2\mathrm{CO}\,$$

4 $${{\rm{K}}}_{2}{{\rm{CO}}}_{3}+2{\rm{C}}\to 2{\rm{K}}+3\mathrm{CO}\,$$

5 $${\rm{C}}+{K}_{2}{\rm{O}}\to 2{\rm{K}}+\mathrm{CO}\,$$

According to the above discussions, the KOH activation mechanisms proposed are (a) Chemical activation by various potassium compounds involves the etching of carbon frameworks due to the redox reactions resulting in the generation of a porous network and (b) the intercalation of the metallic form of potassium into the carbon framework during the activation resulting in the expansion of this framework^12^. Activation followed by washing removes the intercalated metallic as well as other forms of potassium. As a result, the carbon framework remains expanded without reverting to its nonporous structure, and thus, high microporosity is generated. Sodium hydroxide is also widely used as activating agent. According to previous studies, anthracite NaOH activation significantly increases the surface area from 1017 to 2208 m^2^/g and the micropore volume from 0.05 to 0.4 cm^3^/g, with the NaOH to char ratio varying from 1 to 3^13^. However, at high ratios, the micropores enlarged into mesopores, owing to excessive NaOH etching. Moreover, these conventional methods of activation exhibit limitations in controlling the pore size and formation of bottlenecks and/or micropores, typically with a size below 0.7 nm. Contrary to this, one of the interesting synthetic protocol proposed recently involves the condensation and activation of polymer precursors in molten salt medium with these salts playing the role of an alternative solvent as well as porogen for designing porous carbons.

In this context, a direct synthetic strategy is reported for the one step polymerization, carbonization, and activation of the carbon precursors to produce microporous carbons. Two types of carbon materials were prepared at two different temperatures using KOH and a eutectic mixture of KOH-NaOH as the two molten salt media. The resulting carbon materials were evaluated in terms of their ability to serve as gas storage materials.

## Results and Discussion

**Figure 1:**
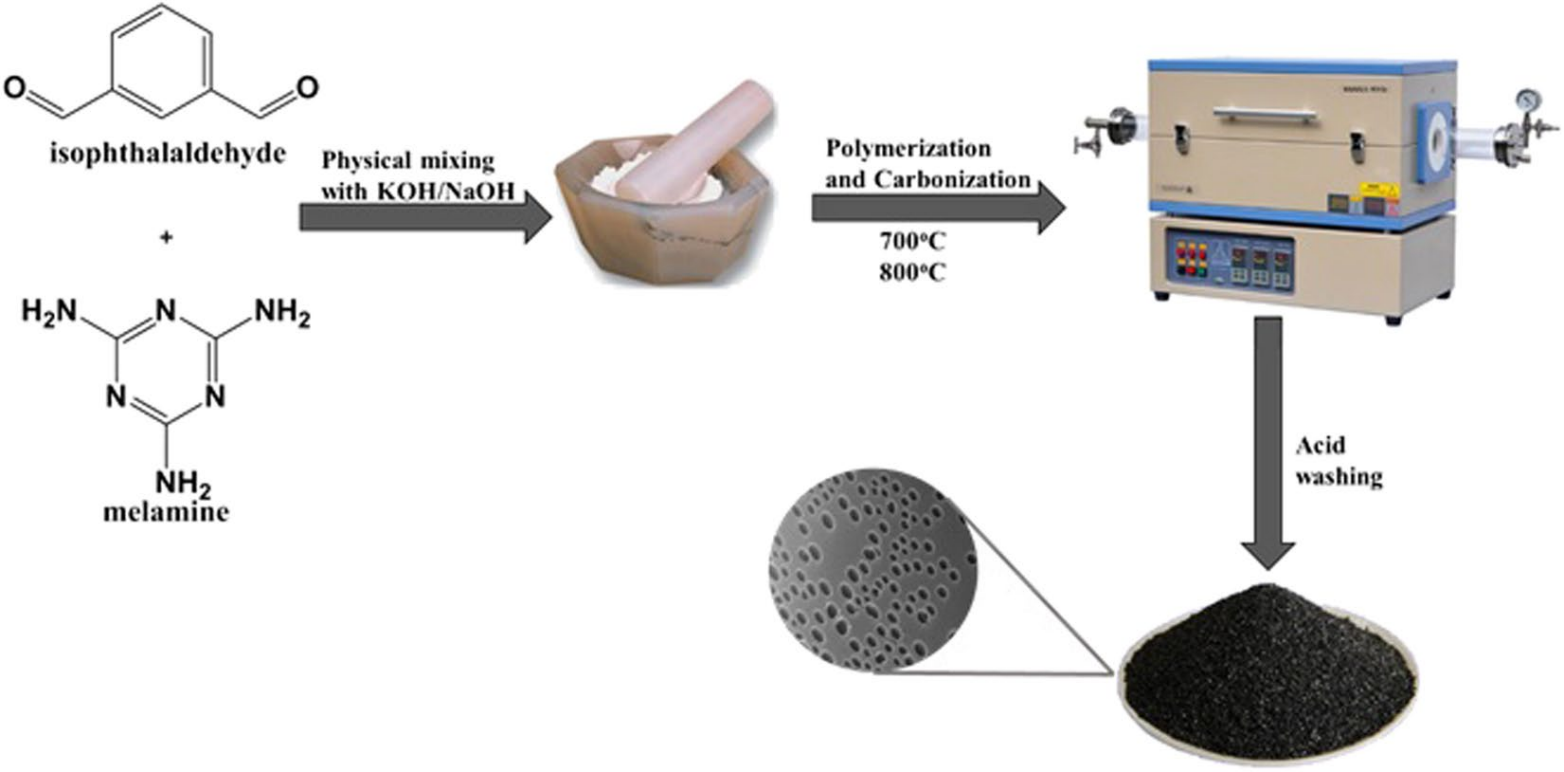
Synthetic procedure for activated microporous carbons.

**Figure 2:**
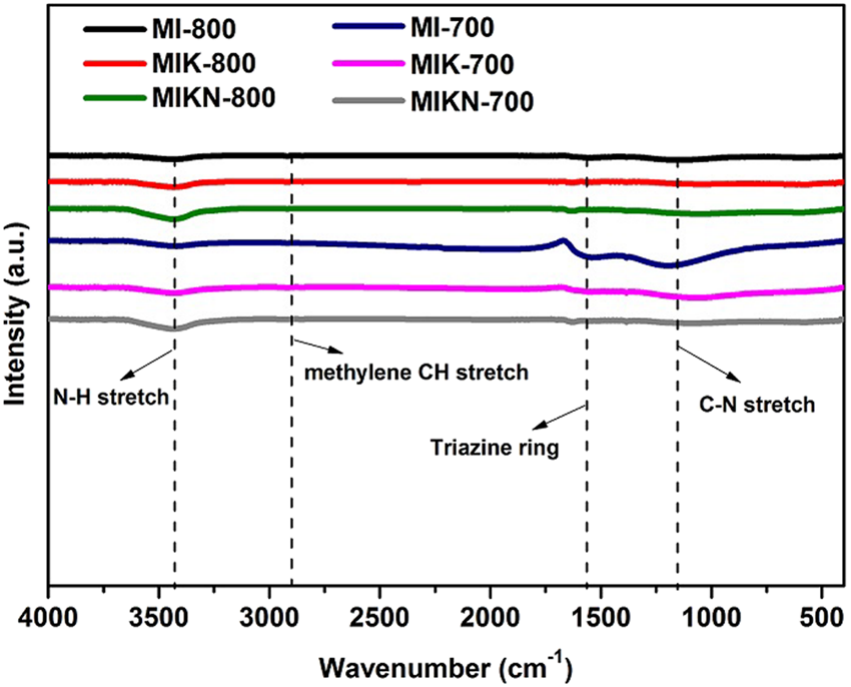
FTIR spectra of fabricated carbon materials.

Porous materials are an emerging platform of scientific interest in the context of their applicability in gas storage, energy storage, and catalytic activities. The successful transformation of the monomers to microporous carbons (Fig. 1) is evident from FTIR spectra. Attenuation of the peak corresponding to the aldehyde at 1690 cm^−1^ as well as the appearance of weak signals at ~3410 cm^−1^ corresponding to the stretching vibration of secondary amine (N−H), and methylene (CH) at 2917 cm^−1^ as the remnants of aminal linkages are confirmation of polymerization of organic precursors (Fig. 2). In addition, the peak of the triazine ring is evident at 1556 cm^−1^. The presence of a broad band at 978–1210 cm^−1^ is ascribed to the vinyl group (=C–H) and (C–N) bonds. The decrease in the intensity of the bands between 1000–1600 cm^−1^ suggest the removal of the O/C–H/C atomic species during carbonization. However, the absence of sharp peaks of any characteristic functionality indicates the effective carbonization of the polymer structure at the targeted temperatures. Moreover, the results indicate the leftovers of nitrogen functionalities which plays a vital role for enhancing CO_2_ adsorption^13^.

**Figure 3:**
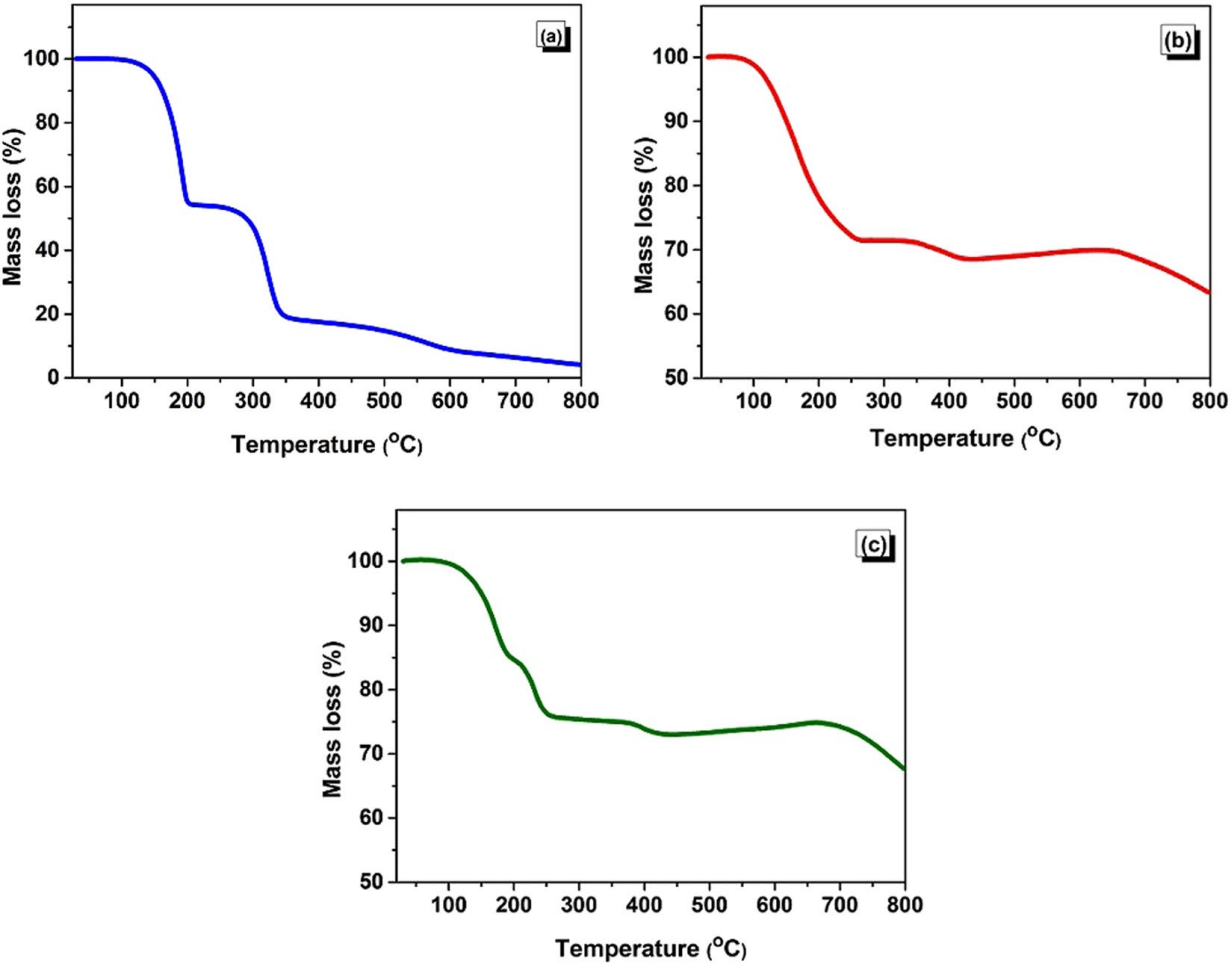
TGA thermograms of (**a**) MI (**b**) MIK and (**c**) MIKN before carbonization.

For a better understanding the effects of the activating agents on the polymerization–carbonization–activation processes, we analyzed the thermal stability of the precursors. Figure 3 presents the thermogravimetric (TG) analysis curves for the three samples (melamine + isophthalaldehyde (MI), melamine + isophthalaldehyde + KOH (MIK), and melamine + isophthalaldehyde + KOH + NaOH (MIKN)). An overlap is observed in the TG curves of the samples with KOH and eutectic KOH–NaOH mixture, indicating that a similar mechanism is involved in the weight loss in the course of one-pot processes. Moreover, it reveals that any dissimilarity in the microstructure of the activated carbonaceous materials are rather due to the physical state of the activating agent during polymerization. Furthermore, eutectic mixture is the physical mixture of NaOH and KOH. The mixture is used for lowering the melting point of individual salt and thus provides a molten medium for monomers to condense at desired polymerization temperature. As anticipated, the sample with this eutectic mixture shows a rapid mass loss at temperatures below 200 °C, which is attributed to its low melting point. However, it should be noted that a strong mass loss that appeared at 346 °C in the TG curve of MI has disappeared in the curves of the sample mixtures with the activating agents. This is justified by the notable evolution of unreacted melamine in MI. Thus, the activating agents act as an efficient reaction media for the *in situ* polymerization in comparison to the hydroxide-free process. Subsequently, a progressive weight loss observed at temperatures above 600 °C is attributed to the carbonization of the polymer. The major component left behind is the carbon with traces of heteroatoms, as proved by elemental analysis. Henceforth, TG curves are used to explore the polymerization–carbonization processes occurring in the eutectic mixtures, resulting in the excellent yield of carbons.

**Figure 4:**
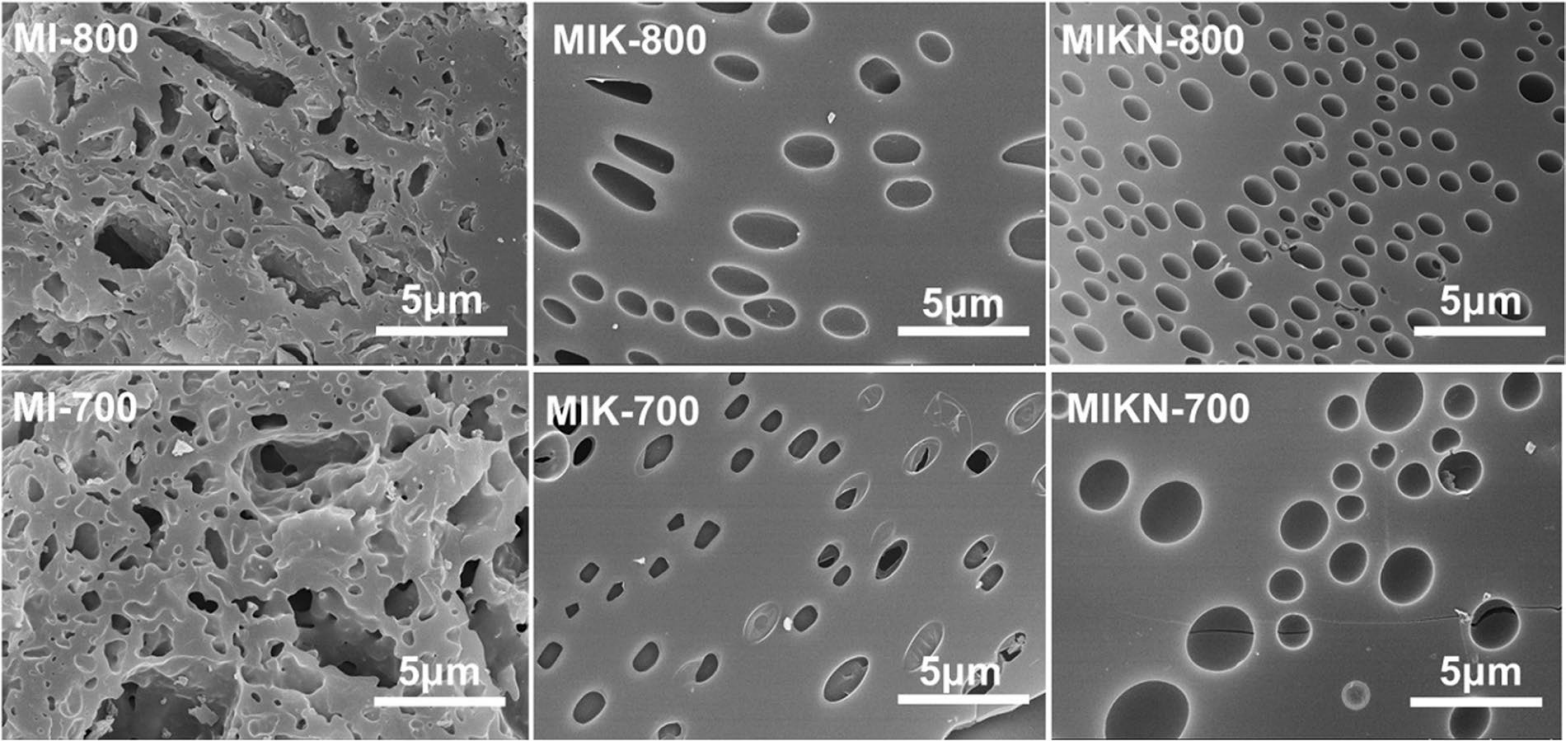
HR-SEM images of fabricated carbon materials.

SEM images **(**Fig. 4**)** clearly show that the carbons fabricated in the presence of hydroxide activating agents exhibit a different morphology than those prepared without them. MI-700 and MI-800 show rough surfaces formed by irregularly interconnected networks with voids, in accordance with their low specific surface areas. However, MIK-700 and MIK-800 exhibit smooth surfaces with some spherical voids. Apparently, polymerization occurred in the liquid media, and the polymer framework has the ability to entrap the generated bubbles released as reaction products or drops of the eutectic mixture. Hence, spherical voids formed in the carbonized material, as observed in the SEM images. Furthermore, MIKN-700 and MIKN-800 exhibit hierarchical porosity with a large number of mesopores. This is attributed to excessive NaOH etching in the eutectic mixture. The diffraction patterns (XRD) of all the carbon materials are presented in Figure S1. From the figure, it is revealed that washing remove all the impurities of sodium and potassium, as no crystalline peak was observed. Consequently, the only peaks observed were attributed to carbon-based materials with the graphitic nature. The diffraction peak of the (002) plane attributed to the periodicity in the ABAB hexagonal close packing of graphitic structures along the z-axis is absent in almost all the diffraction patterns. Apart from this, a high intense scattering peak is observed at low *2θ* values, indicating the disorderness with a low stacking order in graphene sheets, possibly owing to the extensive micropore generation during the activation process.

**Figure 5:**
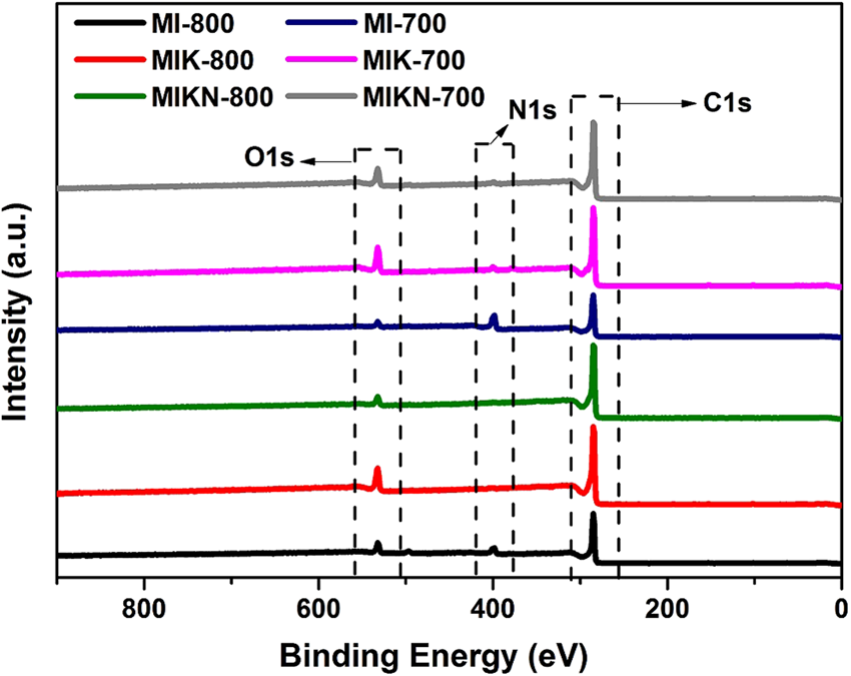
XPS survey scan of adsorbents prepared.

X-ray photoelectron spectroscopy reveals the surface characteristics of prepared carbons. The survey scans **(**Fig. 5**)** show the presence of a high percentage of carbon along with a moderately low oxygen content. The high-carbon content reveals that the prepared sample mainly composed of carbon. The moderate to low nitrogen contents are in accordance with the elemental analysis data. Elemental analysis data (Table 1) indicates that the material contains mainly carbon with a small amount of nitrogen. A careful analysis of the consequences of the carbonization process reveals a large loss of mass with the release of large volumes of volatile gases and some tarry substances^14,15^ and as a result, H and N contents decrease with an increase in the carbon content^16^. All the samples are rich in carbon because the process combined carbonization and activation. The carbon content of MIKN increased gradually from 75.9 to 90.6% as the temperature was increased to 700 to 800 °C, whereas the content of nitrogen decreases from 1.65 to 0.77%. The increase in the carbon content during the carbonization is attributed to two factors: (i) Increased formation of carbon basal planes and (ii) aromatization^17^.

**Table 1:** Elemental composition of the adsorbents prepared.

Materials	Weight (%)		
	C content^a^	H content^b^	N content^c^
MI-800	67.84	0.56	12.57
MIK-800	85.98	0.21	0.76
MIKN-800	90.60	0.22	0.77
MI-700	64.32	0.70	21.65
MIK-700	67.42	0.66	2.66
MIKN-700	75.92	0.42	1.65

^a^Carbon content. ^b^Hydrogen content. ^c^Nitrogen content determined by the elemental analysis.

**Figure 6:**
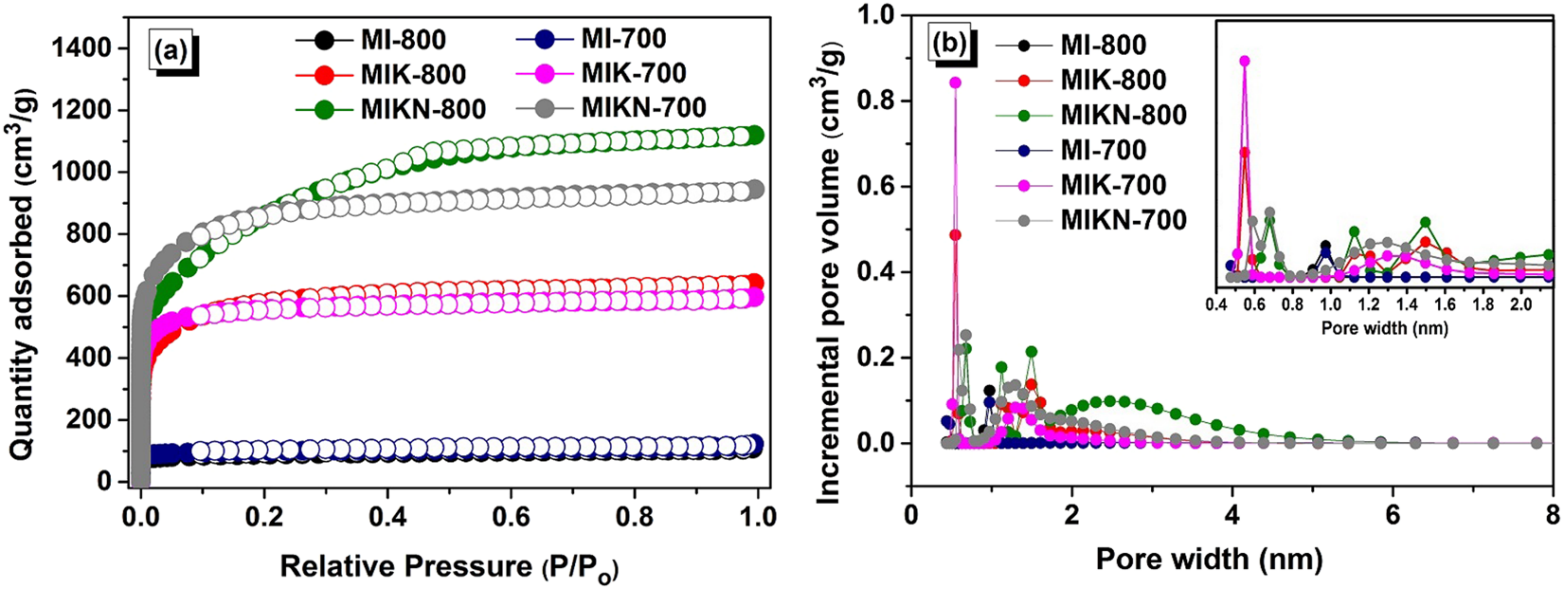
(**a**) Nitrogen adsorption-desorption isotherms and (**b**) pore size distributions of the adsorbents prepared.

Physical properties including the textural features (specific surface area, pore-size distributions, and pore volumes) of the prepared materials were evaluated to assess their role in gas (CO_2_ and H_2_) adsorption. For this purpose, adsorption-desorption isotherms were obtained at 77 K. The isotherms and pore size distributions are presented in Fig. 6(a,b). The textural properties are summarized in Table 2. The adsorption profiles (Fig. 6a) of all the prepared samples indicate rapid uptake of nitrogen gas at low pressures with Type I adsorption characteristic, according to the IUPAC classification^18^. This kind of isotherm indicates a high affinity of the adsorbate and adsorbent for each other, and also suggests that the adsorbent in question is comprised mainly of micropores. However, MIKN-700 and MIKN-800 exhibit both Type I and Type IV isotherms. The isotherms exhibit nitrogen uptake at high pressures, indicating the presence of medium-sized or large mesopores along with the micropores. Furthermore, upon comparison of specific surface areas it is revealed that the initial blank samples, MI-700 and MI-800, prepared without any hydroxide activating agent exhibit a moderately low surface area of 330 and 360 m^2^/g, respectively, which is still better than those of the samples prepared by the old conventional synthesis methods^8,9,19^. Samples synthesized in the presence of KOH only, MIK-700 and MIK-800, possess a high surface area of 2101 and 2090 m^2^/g, respectively. This could be because the KOH activation leads to the generation of micropores primarily, resulting in a higher micropore volume and hence a porous carbon with a higher surface area^20^. On the other hand, samples prepared with the KOH-NaOH mixture, MIKN-700 and MIKN-800, leads to the generation of a hierarchical porous structure with both meso- and micropores with the surface areas of 3246 and 2984 m^2^/g, respectively. It is worth noting that with an increase in the carbonization temperature, hierarchical meso- and microporosity is induced with higher mesopore volume and wider pore size diameter. This can be explained by the decomposition mechanism of salts which upon high temperature reduces to metallic species (sodium and potassium). With the passage of time, a portion of metallic species tends to evaporate from the lattice. However, some proportion of it remains intercalated. Consequently, all these phenomena generate a hierarchical meso- and microporous structure. However, all the superactivated microporous structures were prepared with the mass ratio of 2/1 between the activating agents and monomers. The specific surface area and adsorption profiles of MIK and MIKN are better than those of the materials obtained by chemical activation at high activating agent/precursor ratios^21^. On comparison with the single salt activation method, MIKN-700 exhibit excellent textural features than UAK2–800^20^. Similarly, UANa-960p presents a method with use of 3:1 activating agent to carbon precursor ratio^13^. However, in our work we used lesser amount of activating agents (2:1) with excellent results. Present method generates microporous carbon structure with high specific surface area and narrow pore size diameters. This is accredited to the use of KOH-NaOH eutectic mixture rather than single salt activation. Hence, it is claimed that the present strategy provides a facile way to design highly microporous carbons.

**Table 2:** Textural properties of the adsorbents prepared.

Materials	S_BET_^a^ (m^2^/g)	V_total_^b^ (cm^3^/g)	V_micro_^c^ (cm^3^/g)	V_meso_^d^ (cm^3^/g)	V_micro_/V_total_ (%)	D_pore_^e^ (nm)
MI-800	330	0.20	0.16	0.04	80	6.85
MIK-800	2090	1.31	0.02	1.28	1.5	1.20
MIKN-800	2984	1.98	0.07	1.89	3.5	1.95
MI-700	360	0.26	0.21	0.04	80.7	5.05
MIK-700	2101	1.39	1.33	0.05	95.6	1.05
MIKN-700	3246	1.79	1.58	0.20	88.2	1.24

^a^Surface area calculated by BET method. ^b^Total pore volume determined at P/P_o_ = 0.98. ^c^Micropore volume. ^d^Mesopore volume. ^e^Maxima of the pore size distribution determined by the NLDFT method.

The micropore volumes of the prepared samples were estimated using the Dubinin–Radushkevich (D–R) equation shown below:

$$W={W}_{o}{\exp }[-B(\frac{T}{\beta })Lo{g}^{2}(\frac{P}{{P}_{o}})]$$

where, *W* indicates the amount of the liquid adsorbed at *P/P*_0_, *W*_0_ is the total amount of the adsorbate in the micropores, *B* is the adsorbent constant, and *β* represents the affinity coefficient (0.34 for N_2_)^22^. The ratio of the micropore volume to total volume turns out to be 80, 1.5, 3.5, 80.7, 95.6, and 88.2% for MI-800, MIK-800, MIKN-800, MI-700, MIK-700, and MIKN-700, respectively. The samples prepared at 700 °C possesses a higher micropore volume compared to that prepared at 800 °C, suggesting that the activation at higher temperature is detrimental for the generation of micropores; instead, it stimulates the formation of mesopores. Similar outcomes were observed by Prahas *et al*. who reported that the elevation of the activation temperature drive the widening of the micropores into mesopores^23^.

To investigate the textural parameters deeply, pore size distribution curves were obtained by the nonlocal density functional theory (NLDFT). Figure 6(b) illustrates the pore size distributions of all the prepared samples. The pore size of the samples prepared in the presence of molten salts lies in the range of 0.5 and 3 nm. In contrast, MI possesses mesopores in combination with micropores, and therefore, the average pore size of these samples lies at ~6 nm. It is well known phenomenon that the pore size less than 1 nm is highly beneficial for gas adsorption and storage. Consequently, samples with micropores of an optimum pore size can be employed as efficient candidates for gas adsorption^24^.

**Figure 7:**
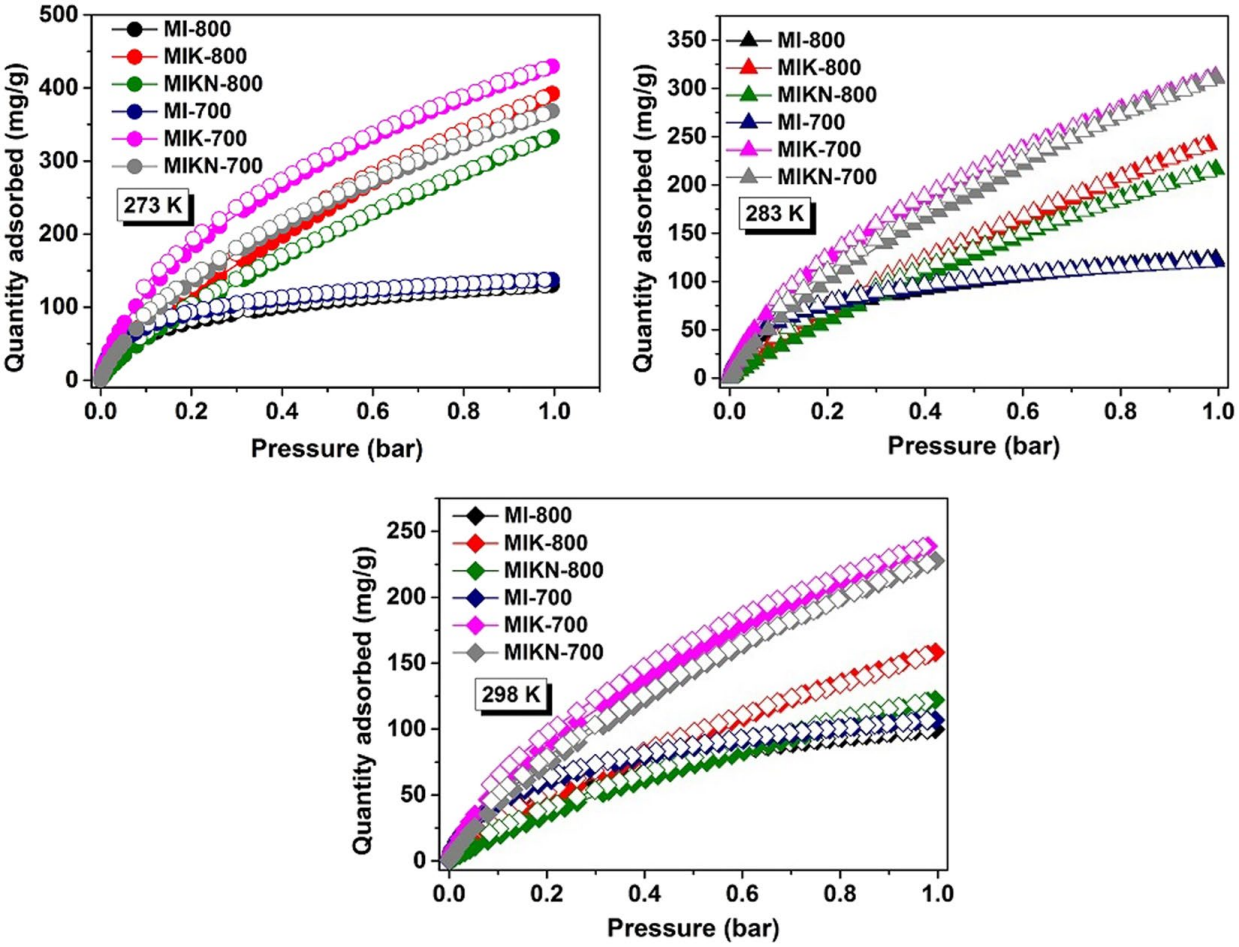
CO_2_ adsorption-desorption isotherms.

At pressures up to 1 bar, the CO_2_ adsorption isotherms obtained at 273, 283, and 298 K, respectively, are presented in Fig. 7 and the complete adsorption data is listed in Table 3. At 1 bar and 273 K, microporous carbons exhibit an excellent CO_2_ uptake of 9.7 mmol/g (429.6 mg/g), 8.9 mmol/g (392.1 mg/g), 8.3 mmol/g (368.4 mg/g), 7.5 mmol/g (333.1 mg/g), 3.1 mmol/g (137.6 mg/g), and 2.9 mmol/g (130.1 mg/g), respectively, for MIK-700, MIK-800, MIKN-700, MIKN-800, MI-700, and MI-800, which are comparable to the CO_2_ uptake of recently reported CO_2_ adsorbents (Table 4). The CO_2_ adsorption isotherms of all the sorbents are nonlinear, indicating their microporous nature. All the prepared samples suffered a clear loss in the CO_2_ uptake with an increase in temperature from 273 to 298 K. This trend is in accordance with the exothermic nature of the adsorption process, implying that the amount of adsorbed CO_2_ decreases with an increase in the temperature^25^. One of the representative samples exhibits a CO_2_ uptake of 9.7 mmol/g (429.6 mg/g) at 273 K which decreases to 7.0 mmol/g (312.2 mg/g) and 5.4 mmol/g (238.7 mg/g) at 283 and 298 K, respectively.

**Table 3:** CO_2_ and H_2_ adsorption capacities along with the isosteric heats of adsorption (Q*st*) for CO_2_ gas determined at 273 and 283 K, shown by the prepared microporous carbons.

Materials	CO_2_ uptake			Q*st* (kJ/mol)	H_2_ uptake wt. (%) 77 K
	273 K (mg/g)	283 K (mg/g)	298 K (mg/g)		
MI-800	130.1	123.4	99.0	34.6	0.95
MIK-800	392.1	343.9	158.2	31.6	3.1
MIKN-800	333.1	216.4	122.2	33.5	4.0
MI-700	137.6	120.7	106.9	37.9	0.98
MIK-700	429.6	312.2	238.7	38.2	2.58
MIKN-700	368.4	310.7	227.7	40.5	3.07

**Table 4:** Comparison of CO_2_ uptake by various adsorbents at 273 K and 1 bar.

Materials	CO_2_ Uptake (mmol/g)	References
**MIK-700**	**9.7**	**Present work**
**MIK-800**	**8.9**	**Present work**
Petroleum pitch	8.6	^48^
Petroleum coke	6.1	^49^
Polypyrrole	4.3	^50^
Bamboo-3-873	7.0	^51^
MAC	6.0	^52^
NC-650-3	7.0	^53^
SNU-C1-va	3.49	^54^
CS-6-CD-8	8.05	^55^
Microporous organic polymer	3.63	^56^
SG–MOP-5	3.37	^57^

Furthermore, the relationship between the CO_2_ adsorption ability and the textural properties of the activated carbons materials was evaluated. From the textural studies it is revealed that the samples prepared in the presence of KOH exhibit well-defined micropores with a higher micropore volume and narrower pore size distribution. More specifically, MIK-700 and MIK-800 exhibit narrow pore sizes, suggesting that the contribution of narrow micropores to the adsorption capacity is greater than those of the wider micropores and mesopores. This is because, in low pressure studies, these narrow micropores are easily filled by the CO_2_ molecules with a greater adsorption potential compared to wider pores. Furthermore, the higher activation temperature (MIKN-800) and presence of NaOH (MIKN-800 and MIKN-700) lead to wider micropores: 1.95 nm for MIKN-800 and 1.79 nm for MIKN-700. Consequently, there is an increase in the specific surface area and pore volume (2984 m^2^/g and 1.98 cm^3^/g for MIKN-800 and 3246 m^2^/g and 1.79 cm^3^/g for MIKN-700, respectively) but they contributed less to CO_2_ adsorption at the atmospheric pressure because no pore filling occurred in these cases. It has been reported that narrow micropores play a crucial role in CO_2_ adsorption^26,27^; however, the correlation between the pore size and CO_2_ adsorption has been rarely investigated. Presser *et al*. reported a perfectly linear relationship between the pore structure (with pore diameter of <0.8 nm) and CO_2_ uptake at 1 bar for carbide-derived carbons^28^. Apart from a suitable texture, MIK-700 also possesses higher nitrogen content (2.66%) compared to other samples prepared in molten salt media. This can improve the Lewis acid-base interactions between CO_2_ molecules and nitrogen moieties, leading to efficient adsorption. Consequently, the results confirm that the fabrication of efficient CO_2_ carbon sorbents requires a precise control of the porosity, with a pore size of approximately 1 nm along with the hetero-atom doping.

**Figure 8:**
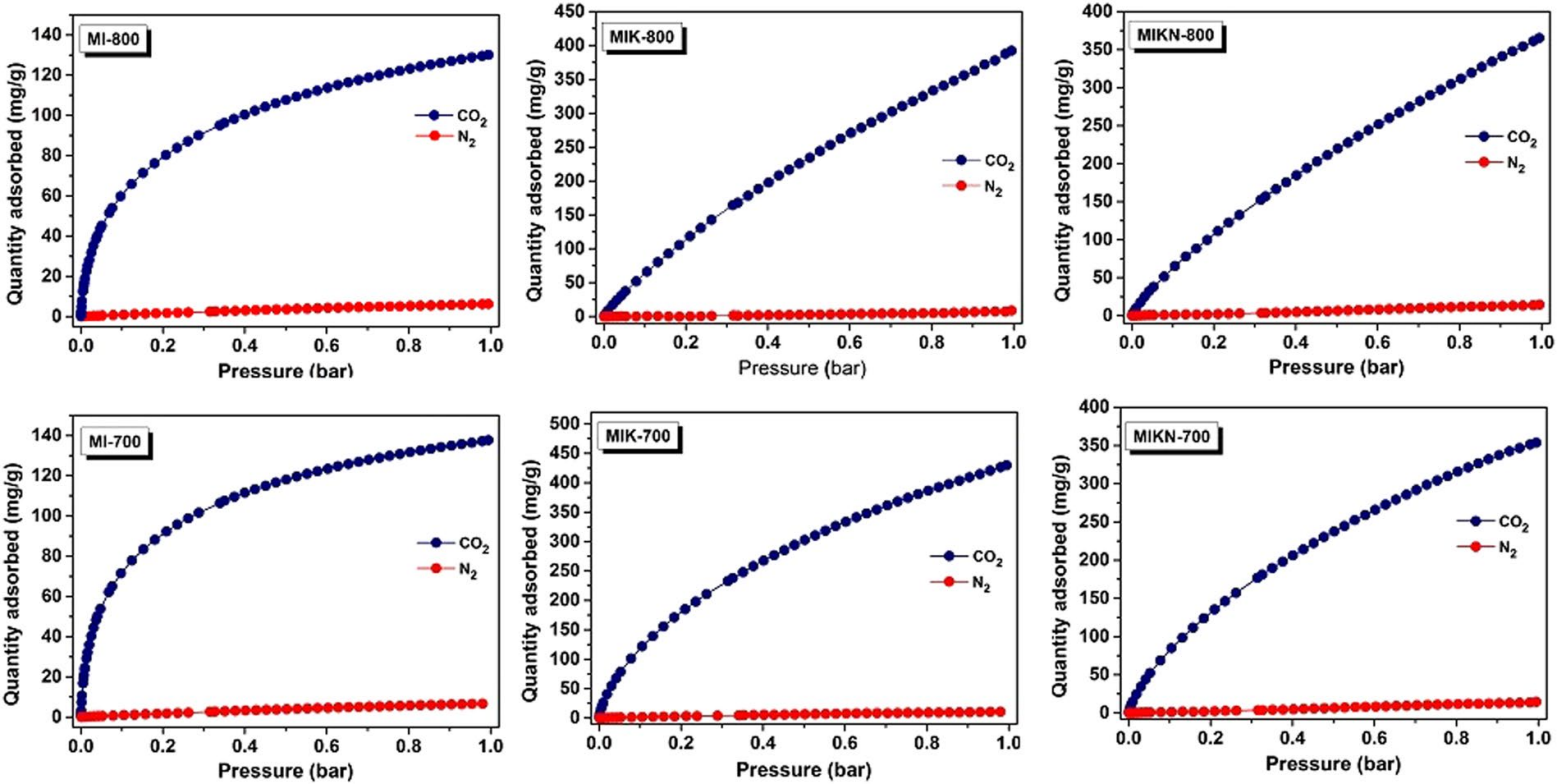
CO_2_ and N_2_ adsorption isotherms obtained at 273 K for selectivity measurements.

Easy regeneration is another critical feature that must be considered while designing CO_2_ sorbents. In this respect, porous carbons offer certain advantages over other materials, such as zeolites. The isosteric heat of adsorption (${\rm{\Delta }}{H}_{ads}\,$or Q*st*) was calculated on the basis of the Clausius−Clapeyron equation shown below.

$$\mathrm{ln}(\frac{{P}_{2}}{{{\rm{P}}}_{1}})=-\frac{{{\rm{\Delta }}{\rm{H}}}_{{\rm{ads}}}}{{\rm{R}}}(\frac{1}{{T}_{2}}-\frac{1}{{T}_{1}})$$

where, *P*_2_ and *P*_1_ are the values of *p*(*CO*_*2*_), *R* denotes the universal gas constant, and *T*_*2*_ and *T*_*1*_ are 273 and 283 K, respectively. Figure S2 shows the *Q*_*st*_ values as a function of adsorbed CO_2_; the corresponding data is listed in the Table 3. The initial values of *Q*_*st*_ are in the range of 31.6–40.5 kJ/mol at a low surface coverage. However, the values show a decreasing trend with an increase in the amount of adsorbed gas, indicating that the interactions between CO_2_ and the pore walls are stronger than those between the CO_2_ molecules. The *Qst* values for the prepared samples are comparable to those of the nitrogen-doped templated carbons from zeolite and mesoporous silica (31−36 kJ/mol)^29,30^, nitrogen-incorporated hierarchical porous carbons (33−37 kJ/mol)^31,32^, and triptycene-derived benzimidazole-linked polymers (29 kJ/mol)^33^. This implies that the adsorption is physical in nature, which strongly depends on the pore structure of porous carbons. If Q*st* is too large, the regeneration of the sorbents becomes too costly, and if it is too small, the CO_2_ uptake and CO_2_ selectivity over N_2_ selectivity will be too small^34^. However, while choosing a suitable adsorbent material, the absolute uptake of CO_2_ is not the only factor to be considered; selectivity over other competing gases and particulates is also an important feature. For a material showing excellent adsorption profile for pure CO_2_ gas but simultaneously adsorbing a large amount of another gas in a more realistic gas mixture, the working capacity would be dramatically reduced. Ideally, CO_2_ adsorbents must exhibit a selective adsorption behavior over all other sorbates. For this reason, CO_2_/N_2_ selectivity studies were performed at 273 K. Selective adsorption behaviors of the adsorbents can be well-explained from the view point of molecular sieving, which demonstrates that kinetic diameters plays a dominant role during the gas adsorption. The kinetic diameters of CO_2_ and N_2_ are 3.30 and 3.64 Å, respectively, as reported earlier^35^. Hence, owing to the small diameter, CO_2_ is adsorbed more than N_2_. Apart from this, another factor to be considered is the thermodynamics, emphasizing the role of the critical temperature of the two gases in the selective adsorption behavior. The critical temperatures (*T*_*c*_) of gaseous CO_2_ and N_2_ are reported to be 304.2 and 126.2 K, respectively. As CO_2_ has a higher critical temperature, CO_2_ molecules easily condense and adsorb on the porous framework. The selectivities for CO_2_ over N_2_ (Fig. 8) determined by initial slope method comes out to be 238, 141, 53, 230, 111, and 71 at 273 K for MI-800, MIK-800, MIKN-800, MI-700, MIK-700, and MIKN-700, respectively, which are comparable to those of highly selective CO_2_ adsorbents^36–38^.

**Figure 9:**
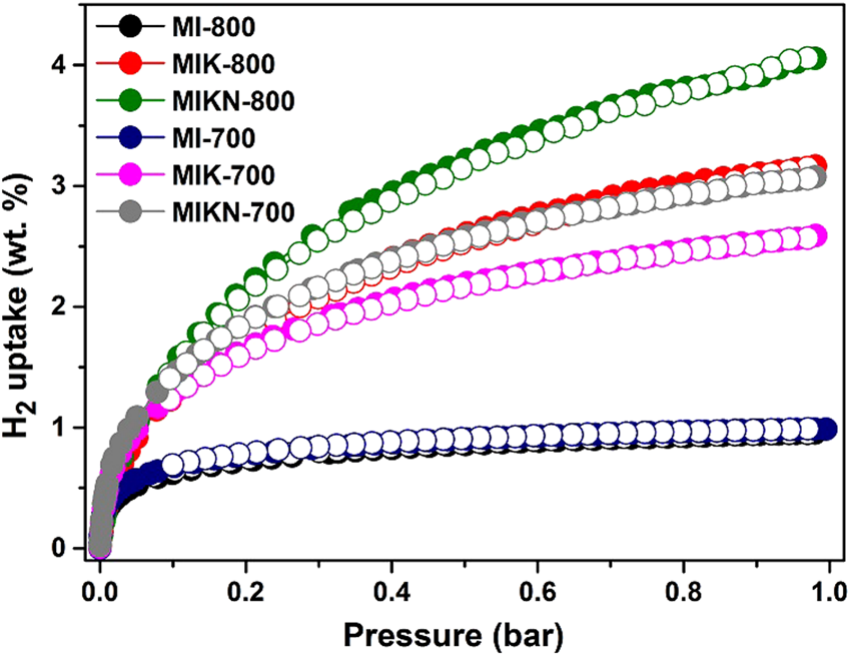
Hydrogen adsorption-desorption isotherms obtained at 77 K up to 1 bar.

The hydrogen adsorption isotherms obtained at 77 K and 1 bar are presented in Fig. 9 and the details are listed in Table 3. The adsorption capacity can be enhanced either by increasing the storage pressure or decreasing the adsorption temperature to 77 K. Here, we chose a low temperature adsorption phenomenon. All the isotherms are Type I (Langmuir-type) isotherms, demonstrating that the formation of a hydrogen monolayer results in a saturation condition which is a usual characteristic for microporous surfaces. However, with an increase in the pressure up to 1 bar, the gas uptake increases continuously but a clear plateau does not appear because of the low pressure measurements. Sample MIKN-800 shows the highest hydrogen storage capacity, reaching up to 4.0 wt% at 1 bar, which is attributed to its highest surface area as well as the maximum pore volume. The present work demonstrates a high performance than the previously reported data, 2.4 wt% for AX21^39^, 2.8 wt% for activated carbons^40^, 2.0 wt% for corncob^41^, 2.6 wt% for wood^42^, and 2.7 wt% for Quercus agrifolia-based ACs^43^. Additionally, MIKN-800 outperforms many MOFs and POPs in terms of its ability to store hydrogen gas at 77 K. This is due to high surface area with optimum pore structure, narrow pore-diameter with hierarchical meso-micro and ultra-micropores as well as nitrogen-doping. Heteroatom doping shows beneficial impact on gas storage property, as already reported in literature^44,45^. Moreover, samples MI-700 and MI-800 have the lowest surface areas and pore volumes and exhibit the lowest H_2_ uptake of 0.95 wt%. Based on these results, we conclude that good correlation exists between the textural features (surface area and pore volume) and gas adsorption capacity of the carbons^46,47^.

## Conclusions

Sustainable microporous carbons were synthesized via a one-step condensation-activation strategy in the presence of molten salts. The hydroxide-containing molten salts are responsible for generating well-developed microporous structures with a high surface area. CO_2_ and H_2_ adsorption properties of the fabricated materials were investigated and the results reveal that the microporous carbons prepared using KOH at 700 °C exhibit an exceptionally high CO_2_ adsorption (uptake = 9.7 mmol/g at 273 K and 1 bar). This outstanding CO_2_ adsorption performance of the prepared carbons is principally attributed to the narrow micropores (1.05 nm) and ultimately the optimum micropore volume (1.33 cm^3^/g). Similarly, the high specific area and pore volume contribute significantly to the H_2_ storage performance at 77 K, with 4.0 wt% uptake of H_2_ by MIKN-800 (specific surface area and pore volume of 2984 m^2^/g and 1.98 cm^3^/g, respectively). Hence, present work contributes an effort to design solvent-free single step strategy for high surface area containing microporous carbons as novel materials for efficient CO_2_ and H_2_ adsorption with an excellent CO_2_/N_2_ selective adsorption behavior.

## Methods

### Materials and synthesis

All the reagents comprising melamine (99%), isophthalaldehyde (99%), sodium hydroxide (98%), potassium hydroxide (95%), and hydrochloric acid were obtained from Sigma–Aldrich. In this study, melamine and isophthalaldehyde were carbonized to design two different types of porous carbons. The first, labeled as MIK-X, was synthesized using 1.24 g of melamine (M) with 1.36 g of isophthalaldehyde (I), and 5.0 g of KOH (K). All the reagents were mechanically ground and heated directly at 250 °C for 180 min followed by carbonization at the targeted temperature for 60 min with the heating rate of 1 °C min^−1^. The resulting product was neutralized to pH 7 by washing sequentially with 3 M HCl solution and distilled water. The obtained material was allowed to dry overnight at 120 °C. The other sample is denoted as MIKN-X. The preparation was carried out following the same procedure described for MIK-X, except that the composition of the activating agents was 2.5 g KOH and 2.5 g NaOH (KN). For evaluating the role of molten salts, blank samples containing a mixture of melamine and isophthalaldehyde (MI-X) were prepared in the absence of the hydroxides. In the names of the prepared samples, X denotes the targeted temperature of carbonization (700 or 800 °C).

### Physicochemical characterization

The synthesized carbon materials were characterized by different analytical techniques. FTIR spectra of the fabricated samples were collected between 4000−400 cm^−1^ using a Fourier transform-infrared vacuum VERTEX 80 V spectrometer. A thermal gravimetric analyzer (TGA; TG209F3) determined the thermal stability of the prepared materials under nitrogen atmosphere. X-ray diffraction patterns were recorded on a D2 PHASER, BRUKER, X-ray diffractometer. All the recordings were made in the range of 2° to 80° (*2θ*). For exploring the morphological structures, field emission scanning electron microscopy (FESEM; Model SU8010, Hitachi Co., Ltd.) was used. Elemental analysis was performed using an EA1112 element analyzer. Further surface characterization was performed by X-ray photoelectron spectroscopy using XPS, VG Scientific Co., ESCA LAB MK-II. The textural features of the prepared materials were determined by obtaining N_2_ adsorption−desorption isotherms from a Model Belsorp Max instrument (BEL Japan, Inc.). Carbon dioxide adsorption capacity at 273, 283, and 298 K and hydrogen storage at 77 K were determined by a Model Belsorp Max instrument (BEL Japan, Inc.). For the CO_2_/N_2_ selectivity measurements, adsorption measurements for both the gases were carried out at 273 K using a Model Belsorp Max instrument (BEL Japan, Inc.). Prior to all the adsorption measurements, the fabricated materials were stored under vacuum conditions by heating at 120 °C for 8 h.

## Electronic supplementary material

Supplementary Information
