# Supplementary material for: Microporous carbons derived from melamine and isophthalaldehyde: One-pot condensation and activation in a molten salt medium for efficient gas adsorption
Source: Sci Rep. 2018 Apr 17;8:6092. doi: 10.1038/s41598-018-24308-z (PMC5904172; doi:10.1038/s41598-018-24308-z)
Supplement: Supplementary file 1 — Supplementary Information [file 41598_2018_24308_MOESM1_ESM.doc]

**Electronic Supplementary Information**

Microporous carbons derived from melamine and isophthalaldehyde: One-pot condensation and activation in a molten salt medium for efficient gas adsorption

Adeela Rehman and Soo-Jin Park^*^

*Department of Chemistry, Inha University, 100 Inharo, Incheon 22212, Korea*

* Corresponding author. Tel.: +82-32-860-8438; Fax: +82-32-860-8438.

*E-mail address:* sjpark@inha.ac.kr (S.-J. Park)

**Figure S1.** XRD patterns of materials prepared.

**Figure S2.** Isosteric heats of adsorption.
